# Malaria transmission-blocking conjugate vaccine in ALFQ adjuvant induces durable functional immune responses in rhesus macaques

**DOI:** 10.1038/s41541-021-00407-3

**Published:** 2021-12-09

**Authors:** Puthupparampil V. Scaria, Charles Anderson, Olga Muratova, Nada Alani, Hung V. Trinh, Steven T. Nadakal, Irfan Zaidi, Lynn Lambert, Zoltan Beck, Emma K. Barnafo, Kelly M. Rausch, Chris Rowe, Beth Chen, Gary R. Matyas, Mangala Rao, Carl R. Alving, David L. Narum, Patrick E. Duffy

**Affiliations:** 1 Laboratory of Malaria Immunology and Vaccinology, NIAID/NIH, 29 Lincoln Drive, Building 29B, Bethesda, MD 20892-2903 USA; 2 U.S. Military HIV Research Program, Walter Reed Army Institute of Research, 503 Robert Grant Avenue, Silver Spring, MD 20910 USA; 3 Henry M. Jackson Foundation for the Advancement of Military Medicine, 6720A Rockledge Drive, Bethesda, MD 20817 USA; 4 Present Address: Pfizer, Vaccine Research and Development, Pearl River, NY USA

**Keywords:** Malaria, Adjuvants

## Abstract

Malaria transmission-blocking vaccines candidates based on Pfs25 and Pfs230 have advanced to clinical studies. Exoprotein A (EPA) conjugate of Pfs25 in Alhydrogel^®^ developed functional immunity in humans, with limited durability. Pfs230 conjugated to EPA (Pfs230D1-EPA) with liposomal adjuvant AS01 is currently in clinical trials in Mali. Studies with these conjugates revealed that non-human primates are better than mice to recapitulate the human immunogenicity and functional activity. Here, we evaluated the effect of ALFQ, a liposomal adjuvant consisting of TLR4 agonist and QS21, on the immunogenicity of Pfs25-EPA and Pfs230D1-EPA in Rhesus macaques. Both conjugates generated strong antibody responses and functional activity after two vaccinations though activity declined rapidly. A third vaccination of Pfs230D1-EPA induced functional activity lasting at least 9 months. Antibody avidity increased with each vaccination and correlated strongly with functional activity. IgG subclass analysis showed induction of Th1 and Th2 subclass antibody levels that correlated with activity.

## Introduction

The WHO malaria vaccine technology roadmap includes the goal to develop and deliver a vaccine that interrupts parasite transmission in mass campaigns for the elimination and ultimate eradication^1^. Malaria transmission-blocking vaccines (TBV) induce antibodies that target antigens expressed by the parasite as it develops in the mosquito midgut following ingestion of an infectious blood meal or that target key mosquito molecules involved in parasite development^2^. TBV antibodies prevent successful sporogony, thereby terminating the malaria lifecycle and, through herd immunity, lower the burden of the human infection and disease.

Several TBV candidates that target pre-fertilization or post-fertilization sexual stage antigens are in development^3–11^. Pfs25, a *P. falciparum* antigen expressed on the surface of zygotes and ookinetes in the mosquito midgut following fertilization, was the first TBV gene sequenced and has been studied most extensively. Recombinant Pfs25 proteins suffer from inherent poor immunogenicity. Early human trials of Pfs25 (or its *P. vivax* orthologue Pvs25) candidates yielded an inadequate immune response or unacceptable reactogenicity associated with the adjuvants used for formulation^12^. Different vaccine delivery strategies including nanoparticle and VLPs have been exploited to enhance Pfs25 immunogenicity^13–16^. In an approach similar to approved polysaccharide conjugate vaccines, we developed a chemical conjugate of Pfs25 with the carrier Exoprotein A (EPA) and examined immunogenicity in preclinical and clinical studies^17–23^. Although preclinical studies in mice and testing in malaria naïve humans showed successful induction of immune responses^17–21,23^, Phase I studies of the Alhydrogel-formulated conjugate in malaria-naïve U.S. adults as well as malaria-experienced adults in Mali required four doses to achieve transmission reducing activity and the antibodies generated were of limited duration^23,24^. Thus, studies using this antigen have been encouraging, however vaccine improvements will be required to obtain a sustained and robust immune response.

More recently, attention has shifted to another candidate antigen, Pfs230, expressed on the gametocyte and gamete surface before fertilization. Monoclonal antibodies raised against gametes recognized a 230 kDa protein expressed on its surface and demonstrated transmission-blocking activity in a complement dependent manner^25^. Various regions of this protein have been tested as vaccine candidates since the full-length cysteine-rich rich protein comprises a large complex structure that is difficult to express in heterologous systems^26,27^. Transmission reducing activity of this protein has been mapped to its N-terminal region including the first 6-cysteine domain (domain 1)^28,29^. Various N-terminal fragments of this protein that encompass domain 1 have been tested using different antigen delivery technologies in mouse and rabbit models as vaccine candidates^9,29–34^. We developed a recombinant protein based on domain 1 of Pfs230 in *Pichia pastoris* (amino acid boundaries from Ser^542^- Gly^736^) amenable to large scale manufacturing^28^. This antigen, Pfs230D1, when conjugated with EPA and other carriers showed enhanced immunogenicity and transmission-blocking functional activity in mice, compared to monomer antigen^20,32^. A comparison of Alhydrogel^®^ formulated EPA conjugates of Pfs230D1 and Pfs25 in malaria naïve humans demonstrated a superior transmission-blocking activity by Pfs230D1 compared to Pfs25^35^. Interestingly, the difference in functional activity between these two antigens observed in human studies is more clearly recapitulated in immunogenicity studies in non-human primates than in mice, indicating non-human primate as a better model to study transmission-blocking vaccines^35^. Currently, these conjugates are being evaluated in malaria-exposed humans in Mali (ClinicalTrials.gov IDs: NCT02334462; NCT02942277; NCT03917654). Nevertheless, none of the vaccine candidates based on Pfs230 antigen have been studied extensively in non-human primate models.

Studies in mice have indicated that more potent adjuvants may contribute to a stronger functional immune response against TBV^36^. Adjuvants play a critical role to promote the adaptive immune response^37,38^. Adjuvants designed to target innate pattern recognition receptors (PRR) such as TLRs, have been approved for use in licensed vaccines to induce strong and durable immune responses^39–42^. Based on our clinical studies to date, alum-based adjuvants are not likely to achieve the high and durable antibody responses needed for TBV field efficacy. The WRAIR Laboratory of Adjuvant and Antigen Research has developed the Army Liposome Formulation (ALF), which is a liposomal adjuvant containing synthetic monophosphoryl lipid A, 3D-PHAD^®^, a TLR4 agonist. ALF when formulated with QS-21 (*Quillaja saponaria* Molina bark extract fraction 21) incorporated into the lipid membrane is referred to as ALFQ^43^. ALFQ has been shown to be safe in preclinical models for a PfCSP-based vaccine^44^ and is currently undergoing Phase 1 testing for safety and immunogenicity in healthy human volunteers with two malaria anitgens, FMP013 (ClinicalTrials.gov Identifier: NCT04268420) and FMP014 (ClinicalTrials.gov Identifier: NCT04296279) and a COVID vaccine (ClinicalTrials.gov Identifier: NCT04784767).

In the present study, we evaluated the immunogenicity of EPA conjugates of Pfs25 and Pfs230D1, formulated with ALFQ, in Rhesus macaques. Though Pfs25-EPA and Pfs230D1-EPA formulated in ALFQ gave similar levels of antibody titer in immunized animals, Pfs230D1-EPA showed superior functional activity in standard membrane feed assay, similar to studies of Pfs25-EPA and Pfs230D1-EPA in humans using the adjuvant Alhydrogel^®^. The high functional activity induced by the Pfs230D1-EPA conjugate persisted for at least 9 months after a third vaccination.

## Results

### Immunogenicity and functional activity

**Fig. 1:**
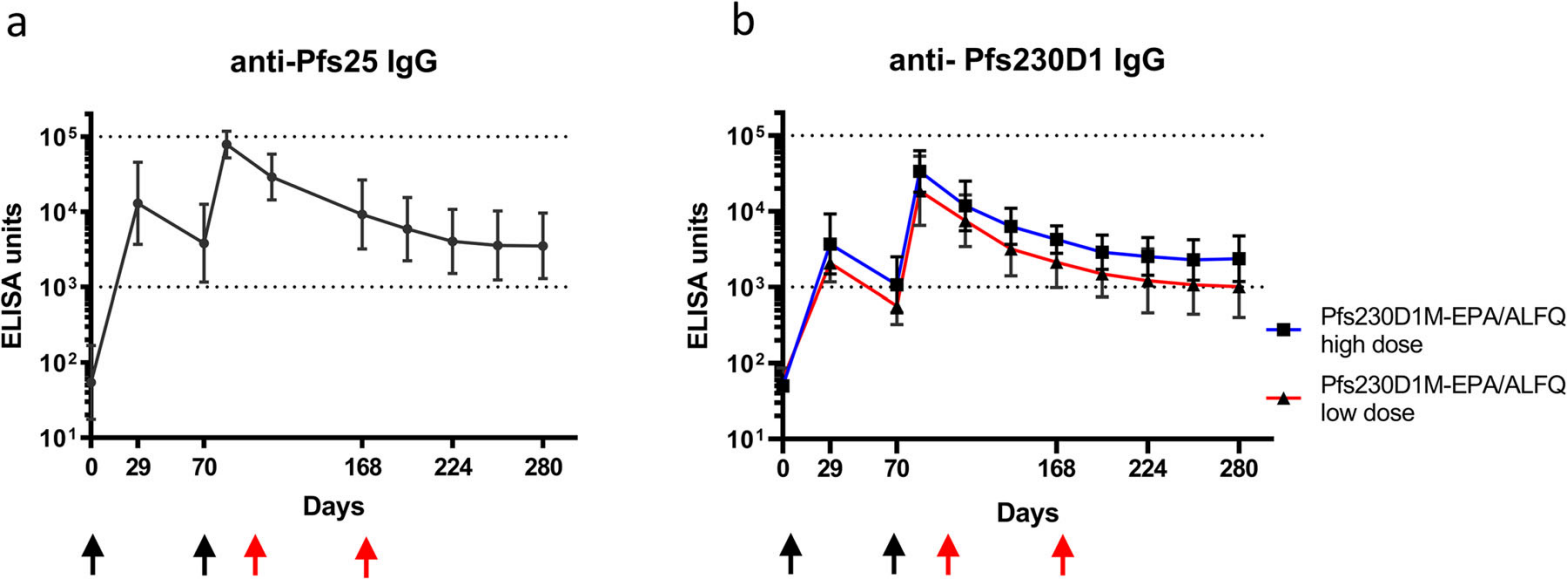
Antibody levels following vaccination with Pfs25-EPA or Pfs230D1-EPA with ALFQ adjuvant. Serum ELISA units of Rhesus monkeys (*n* = 5) vaccinated with Pfs25-EPA (**a**) or Pfs230D1-EPA (**b**) formulated with ALFQ adjuvant. Sera from each animals were analyzed in triplicate, and the y-axis represents the geometric mean of ELISA units with 95% confidence interval. Black arrows indicate the days of vaccination; red arrows indicate the days of SMFA. EU for the high-dose group of Pfs230D1-EPA was significantly higher than that for the low-dose group; *p* = 0.032 by GEE analysis.

Immunogenicity of Pfs230D1-EPA and Pfs25-EPA was evaluated in Rhesus macaques. Groups (*n* = 5/grp) of Rhesus macaques were immunized by intramuscular injection with an antigen dose of 40 µg or 9.7 µg (subsequently referred to as “high dose” or “low dose” respectively) of Pfs230D1-EPA or 47 µg (referred to as “high dose”) of Pfs25-EPA, all formulated with ALFQ adjuvant. Dose refers to the amount of Pfs230D1 or Pfs25 in the respective conjugates. The “high” doses for each antigen correspond to the highest dose evaluated in clinical trials. Animals were vaccinated on days 0 and 70, to be consistent with our previous NHP study of Pfs25-OMPC conjugate^45^. This schedule also approximate the initial two vaccinations (0 and 2 months) of the Pfs25-EPA trial conducted in humans^23^. Antibody levels, monitored by ELISA after vaccination, showed induction of strong antigen-specific antibody response by both conjugates against their respective antigens on Day 84 (Fig. 1a, b). Thereafter, antibody levels decreased up to the last measurement on day 280 with an antibody decay half-life of 19 days for Pfs25-EPA and 17 days for both high and low dose Pfs230D1-EPA groups. Although geometric mean antibody levels for both Pfs230D1 conjugate groups gradually decreased, the high dose group maintained significantly higher antibody levels compared to the low dose group. (*p* = 0.032; generalized estimating equation (GEE) model).

**Fig. 2:**
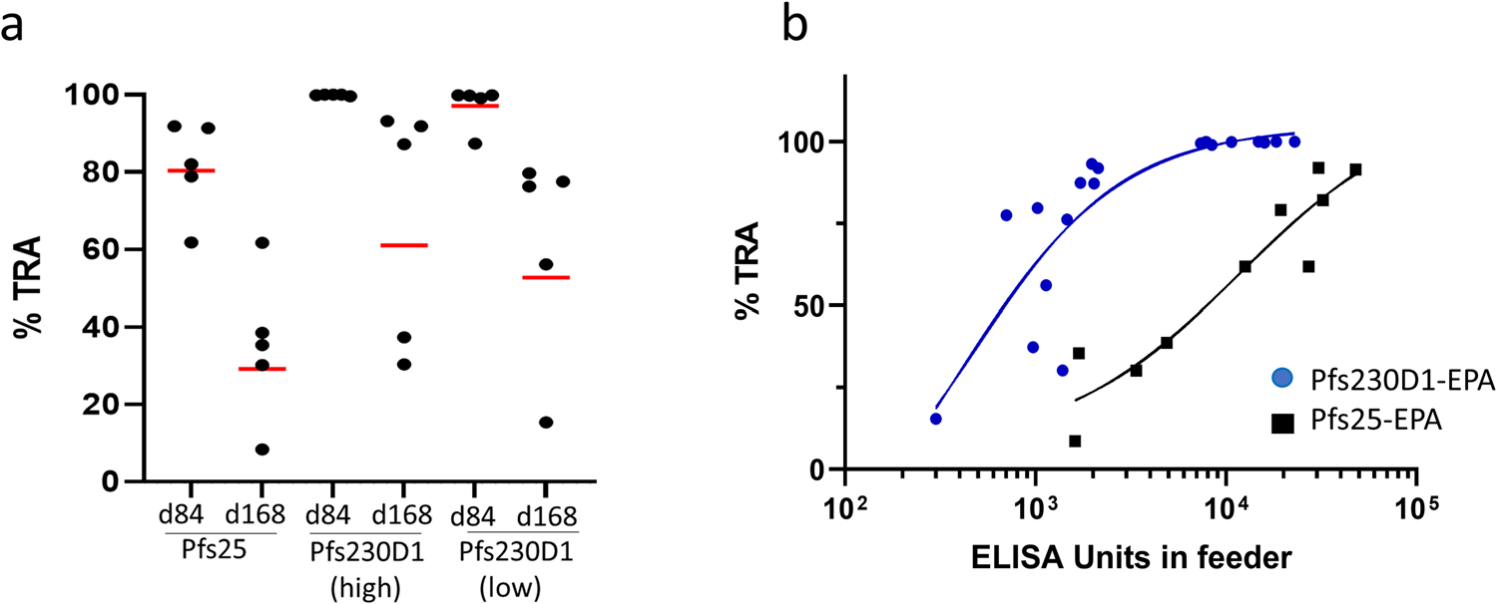
anti-Pfs230 induces superior functional activity than anti-Pfs25 after two immunizations. **a** transmission reducing activity, by group, 2 weeks (d84) and 14 weeks (d168) after dose 2. Each data point represents TRA from one monkey; red lines denote arithmetic mean TRA. **b** Nonlinear 4-parameter curve fit of log (EU) vs TRA. Antibody levels shown account for dilution of immune monkey sera in the feeding assay. An IC_50_ was estimated for both antigens using a 4-parameter curve fit of the log(titer) vs %TRA for each monkey of the d84 and d168 SMFA data combined. Correlation analysis of ELISA vs TRA showed strong correlation for both antigens. Pfs230D1: Spearman *r* = 0.92, *p* < 0.0001; Pfs25: Spearman *r* = 0.94, *p* < 0.0001.

Serum functional activity was assayed by the standard membrane feeding assay (SMFA) using samples collected on days 84 (peak antibody titers) and 168 (Fig. 2a and Supplementary Table 1). On day 84, Pfs230D1 antisera from both dosing groups induced strong inhibition of mosquito infection, with a group mean average transmission reducing activity (TRA, or % reduction in mean parasite count in mosquitoes) of 99.9% and 97.2% for the high and low dose groups, respectively. Among all the animals immunized with Pfs230D1-EPA, 9/10 animals showed >99% TRA at peak antibody levels (Fig. 1). Three animals in the high dose group gave a transmission-blocking activity (TBA, or % reduction in infected mosquitoes) of 100% while the other two gave at least 90% TBA. In the low dose group, TRA was >99% except for one animal which gave a TRA of 87.4%. In this group, 3/5 animals gave a TBA of >95% though none with 100% TBA. In contrast, serum functional activity of animals vaccinated with Pfs25-EPA was substantially lower. On day 84, average TRA was 81.2% and only 2/5 exceeded 90% TRA. TBA for this group was substantially lower than Pfs230D1 groups, ranging from 9.1% to 40.9%.

Sera collected on day 168 (14 weeks after last vaccine dose) also were assayed for functional activity. Consistent with the decrease in antibody levels, functional activity of the immune sera had also decreased. Mean TRA for Pfs230D1-EPA were 68.0% and 61.0% in the high and low dose groups respectively. Three of the five monkeys immunized with Pfs230D1-EPA high dose had >80% TRA whereas none in the low dose group reached 80% TRA. TBA observed was also very low for both groups, ranging from 0% to 22.7% (Supplementary Table 1). Among Pfs25-EPA immunized animals, only 1/5 had greater than 50%TRA and none had any significant TBA on day 168.

### Correlation between antibody titer and functional activity

To assess the correlation between antibody levels and functional activity, ELISA measurements were plotted against TRA for all samples. For both antigens, ELISA units correlated strongly with TRA (Pfs230D1: Spearman *r* = 0.92, *p* < 0.0001; Pfs25: Spearman *r* = 0.94, *p* < 0.0001) (Fig. 2b). A 4-parameter dose-response curve was fit to the data to estimate the IC_50_ for both antigens. The IC_50_ for both Pfs230D1 groups combined was estimated at 502 ELISA units (*R*^2^ = 0.70), whereas the IC_50_ for Pfs25 was estimated at 12,316 ELISA units (*R*^2^ = 0.89), indicating that Pfs230D1-EPA conjugate provides superior functional activity compared to Pfs25-EPA.

### Effect of third vaccination

Even though Pfs230D1-EPA gave superior functional activity compared to Pfs25-EPA, its duration of activity may still be insufficient for an effective transmission-blocking vaccine requiring at least 80% TRA. We explored the possibility of achieving this by a third vaccination. Since the functional activity of Pfs25-EPA was found to be substantially lower compared to Pfs230D1-EPA, we did not pursue Pfs25-EPA for further evaluation.

**Fig. 3:**
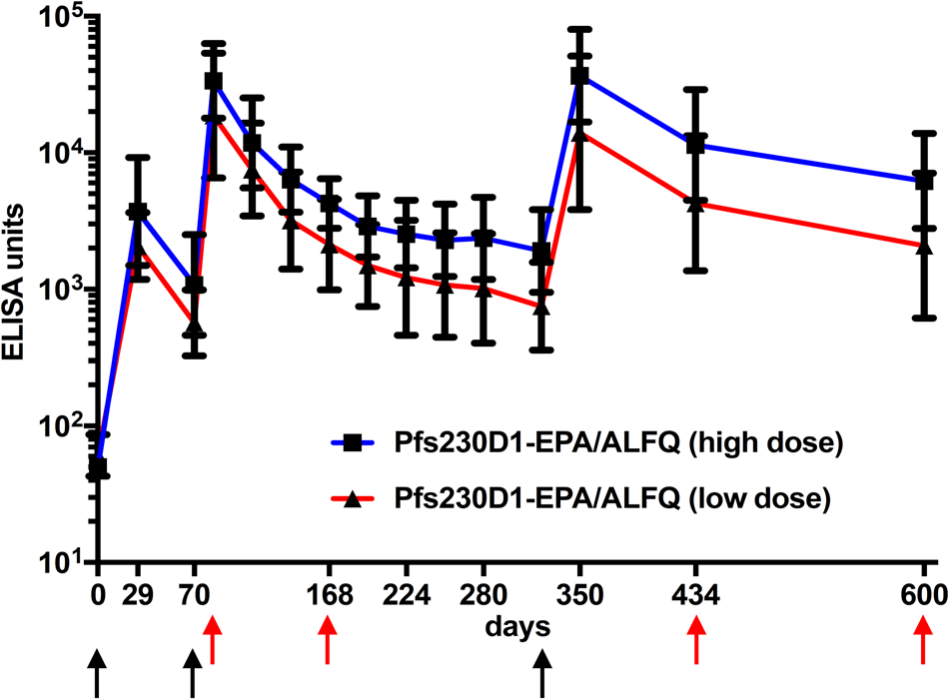
Antibody titers following vaccination with Pfs230D1-EPA at two doses (high or low) formulated with ALFQ adjuvant. Groups of five Rhesus monkeys were vaccinated with the indicated antigen/adjuvant formulations. Black arrows indicate days of vaccination; red arrows indicate days of SMFA. Sera from each animals were analyzed by ELISA in triplicate and the *y*-axis represents the geometric mean of ELISA units with 95% confidence interval. EU for high dose group of Pfs230D1-EPA was significantly higher than that of low dose group; *p* = 0.024 by GEE.

Once the decay of antibody levels after 2nd vaccination stabilized, animals that received Pfs230D1-EPA on days 0 and 70 were administered a 3rd vaccination of Pfs230D1-EPA/ALFQ on study day 322 using the same doses given at the first and second vaccinations, and thereafter followed until day 600. The high dose group maintained a significantly higher antibody levels compared to low dose group until day 600 (*p* = 0.024 by GEE model). On day 350 (4 weeks post 3rd vaccination), geometric mean antibody levels observed were not different from those observed post 2nd vaccination (Fig. 3). Sera from days 434 and 600 were analyzedd to determine the duration for which antibody levels were maintained above a functional threshold. Interestingly, antibody durability was greater after the 3rd vaccination than the 2nd vaccination, for both high and low doses of Pfs230D1 conjugates. For example, from day 84 (peak after 2nd vaccination) to day 322 (day of 3rd vaccination), a period of 238 days, the geometric mean ELISA units for high and low dose groups decreased by 17.6- and 24.9-fold, respectively from the peak titer (Supplementary Table 2). However, from day 350 (peak after 3rd vaccination) to day 600, a period of 250 days, geometric mean ELISA units for high and low dose groups declined by only 5.9- and 6.7-fold, respectively, indicating a slower decline after third vaccination. Antibody decay after 2nd and 3rd vaccinations, analyzed using an exponential model with one phase decay, appears to confirm the slow decline in antibody levels after 3rd vaccination (Supplementary Fig. 3). Antibody decay half-life for the high dose group increased from 17.6 days following the 2nd vaccination (*R*^2^: 0.7733; 95% CI: 10.7–26.9 days) to 36.1 days following the 3rd vaccination (*R*^2^:0.6211; 95% CI: not defined). For the low dose group 17.8-day half life after 2nd vaccination (*R*^2^:0.7221; 95% CI: 10.0–29.0 days) increased to 33.3 days after 3rd vaccination (*R*^2^: 0.3311; 95% CI: not defined). Accuracy of the estimated half-life after 3rd vaccination may be limited by the lack of sufficient number of data points following this vaccination. The apparent increase in half life indicates that the 3rd vaccination may have induced a qualitative difference in the antibody response.

**Fig. 4:**
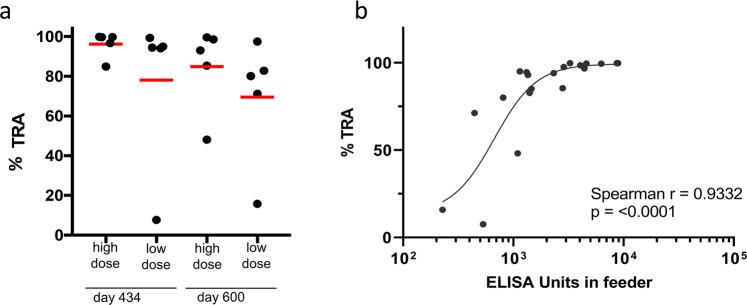
A third vaccination of Pfs230D1-EPA induces durable functional activity. **a** transmission reducing activity, by group, 16 weeks (d434) and 40 weeks (d600) after 3rd vaccination. Each data point represents TRA from one monkey; red lines denote arithmetic mean TRA. **b** nonlinear 4-parameter curve fit of log (titer) vs TRA. Antibody titers shown account for dilution of immune monkey sera in the feeding assay. An IC50 was estimated for using a 4-parameter curve fit of the log(titer) vs %TRA for each monkey of the d434 and d600 SMFA data combined.

To confirm that the sustained antibody levels post third vaccination translated to function, SMFA was performed on sera from days 434 and 600 (16 and 40-week post 3rd vaccination, respectively) (Fig. 4a). On day 434, high dose group gave a mean TRA of 96.2% with 3/5 animals giving >99% TRA and TBA > 80%. The low dose group on day 434 had a mean TRA of 78.1% with 4/5 animals with >90% TRA and none with TBA > 80% (Supplementary Table 3). This group had a TBA ranging from 4.3–73.9%. On day 600, 40 weeks post 3rd vaccination, the high dose group had a mean TRA of 85% and 4/5 animals had >80% TRA. TBA for this group ranged from 4.5 to 82.6. The low dose group on day 600 had a mean TRA of 69.5% with 3/5 animals with >80% TRA (TBA ranged from 0 to 50%). Together, between the two groups, on day 600, 7/10 animals gave >80% TRA. (Fig. 4a and Supplementary Table 3). Therefore, strong functional activity was retained over 9 months after the 3rd vaccination.

A plot of ELISA units vs TRA post 3rd vaccination shows that antibody levels correlated strongly with functional activity (spearman *r* = 0.90, *p* < 0.0001) (Fig. 4b). Comparing ELISA units vs TRA between post vaccination 2 and post vaccination 3, there did not appear to be a difference. The estimated IC_50_ for combined data of days 434 and 600 was 671 ELISA units, similar to what is observed for post vaccination 2 (502 ELISA units).

**Fig. 5:**
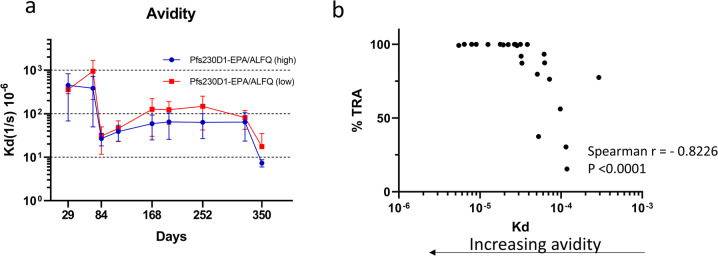
Avidity increased with repeated vaccinations. **a** Antibody avidity, presented as dissociation constant KD, of sera from animals immunized with high (blue) or low (red) dose of the Pfs230D1-EPA conjugate at different time points during the study. Group size = 5. Each point represents the mean value for the group and error bars represent the standard deviation. **b** Correlation between antibody avidity and functional activity (%TRA) of sera from day 84, d168 and d350. Points represent KD for each monkey. Spearman *r* = −0.8226; *p* < 0.0001.

Repeat or booster vaccination has been shown to generate antibody with increased avidity^46,47^, therefore we investigated the effect of repeat vaccinations on the avidity of antibody generated by the Pfs230D1 conjugate in ALFQ. Surface Plasmon Resonance (SPR) technique has been found useful in the measurement of antibody avidity of immune sera^48–51^. We have carried out antibody avidity measurements using sera collected from each animal at various timepoints throughout the study. Figure 5a shows the change in avidity for both high and low dose groups during the study, plotted in terms of dissociation constant *K*_D_. A large increase in avidity (decrease in off-rate) could be seen following the 2nd vaccination (day 29 vs day 84), with the average off-rate decreasing from a *K*_D_ of 34.5 × 10^−5^/s on day 29 to 2.66 × 10^−5^/s on day 84 for the high dose group (Supplementary Table 4). A similar decrease was observed for the low dose group from 35.8 × 10^−5^/s on day 29 to 2.73 × 10^−5^/s on day 84. A further reduction in the off-rate (increase in avidity) was observed for both groups after 3^rd^ vaccination, measured on day 350 (Fig. 5a). A correlation analysis of data from day 84, day 168, and day 350 (days on which both avidity and TRA are available) showed a strong correlation between antibody avidity and functional activity of the sera (Fig. 5b). Sera with high avidity (low *K*_D_) gave high transmission reducing activity. Comparison of off-rates on day 84 (post dose vaccination 2) and day 350 (post vaccination 3) showed that the off-rate decreased from 2.66 × 10^−5^/s to 7.24 × 10^−6^/s for the high dose group (Supplementary Fig. 4a) and from 2.73 × 10^−5^/s to 1.75 × 10^−5^ for the low dose group (Supplementary Fig. 4b), indicating an increase in avidity with repeat administration of the vaccine. Although there appeared to be an increase in avidity, neither group showed a statistically significant difference in avidity between post 2nd and 3rd vaccinations. Both high and low-dose groups gave a *p* value of 0.0625, by Wilcoxon matched-pairs signed rank test, for post 2nd dose vs 3rd dose (day 84 vs day 350).

### IgG subclass analysis

**Fig. 6:**
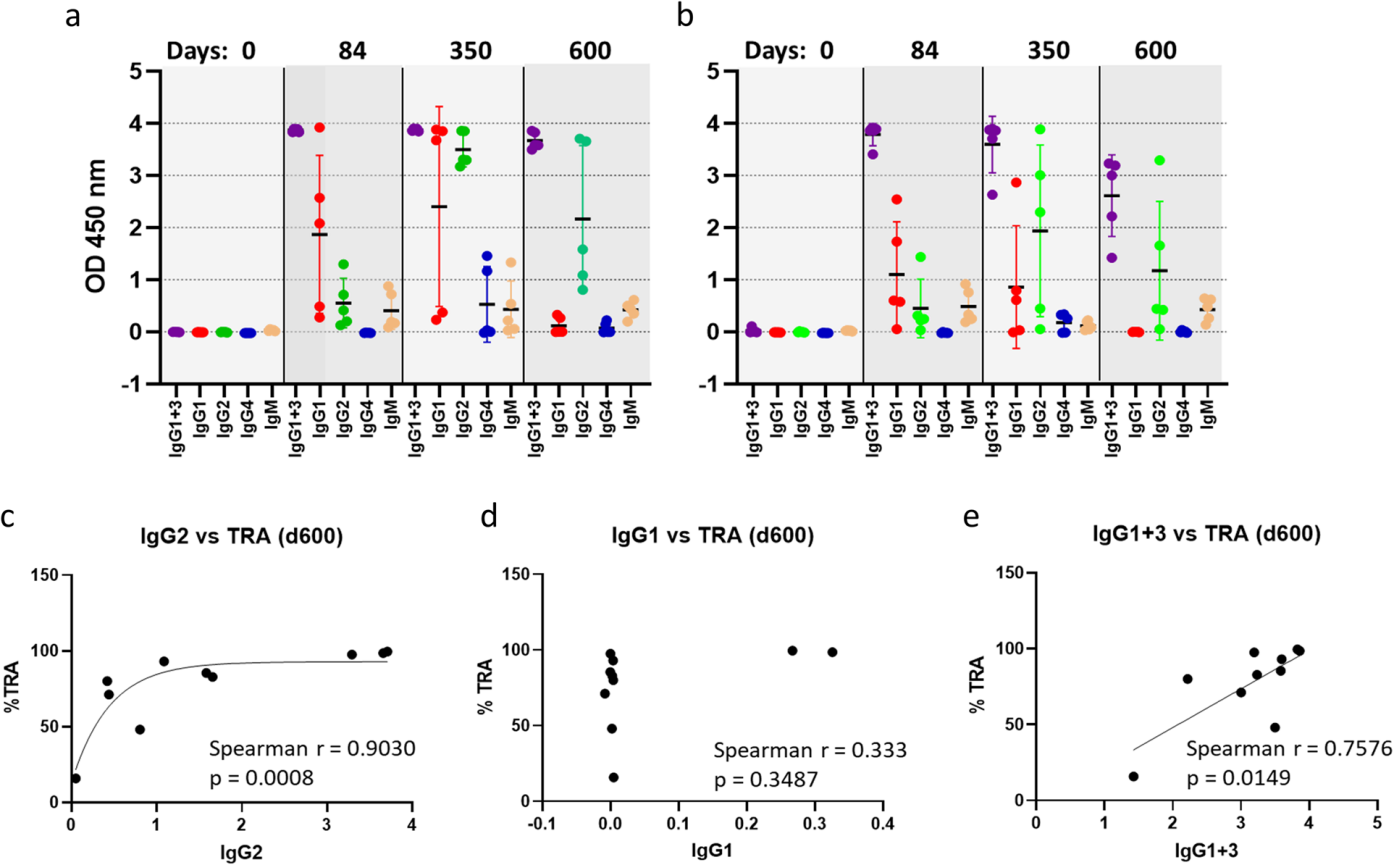
IgG subclass distribution in the immune sera. IgG subclasses (IgG1+3, IgG1, IgG2, IgG4) and IgM antibody levels in sera from Rhesus vaccinated with high dose (**a**) and low dose (**b**) of the Pfs230D1-EPA conjugate formulated with ALFQ assayed at different time points (days 0, 84, 350 and 600) during the study. Error bars represent the mean and standard deviation. Correlation between the functional activity (%TRA) of sera from day 600 and levels of IgG subclasses IgG2 (**c**), IgG1 (**d**) and IgG1+3 (**e**). Immune sera from each animal were analyzed by ELISA using the Rhesus specific monoclonal antibody isotype controls listed in Supplementary Table 5.

Transmission-blocking activity of antibody against Pfs230 is known to be dependent on antibody mediated complement fixation^25,35^. Hence Pfs230 vaccines that generate antibody subclasses that facilitate complement activation may provide higher functional activity. Adjuvants have been shown to alter the IgG subclass distribution affecting the functional activity of Pfs230 conjugates^32,36^. Earlier studies in mice showed induction of IgG2, that facilitate complement activation in mice, resulted in superior functional activity for Pfs230 conjugate vaccines^32^. Pfs230-EPA conjugate formulated in Alhydrogel^®^ generated complement fixing IgG1 in humans and Rhesus and showed transmission-blocking functional activity^35^. Therefore, we analyzed the IgG subclass distribution of Pfs230D1-EPA/ALFQ at different time points to evaluate its role in the transmission-blocking activity of this vaccine. Specifically, we looked for changes in IgG subclass distribution after each vaccination to see if changes in the IgG subclass profile explains the more durable functional activity observed after third vaccination. Figure 6 shows the serum IgG subclass distribution of high (Fig. 6a) and low-dose (Fig. 6b) groups on day 0 (pre-vaccination), day 84 (2 weeks post 2nd vaccination), day 350 (4 weeks post 3rd vaccination), and day 600 (40 weeks post 3rd vaccination). Day 0 sera showed no Pfs230D1 specific antibody response, as expected. IgG1 and IgG3 were assayed together using a secondary antibody that binds to both. Due to lack of IgG3 specific anti-Rhesus secondary antibody, level of IgG3 could not be assayed independently.

For the high-dose group, on day 84 (post 2nd vaccination), the dominant subclasses were IgG1 and IgG3. However, this changed dramatically with the 3rd vaccination, resulting in a substantial increase in IgG2 levels on Day 350. IgG4 and IgM levels were comparatively low at these time points. IgG1 showed substantial variability within the group at both time points. Interestingly, on day 600 (40 weeks post 3rd vaccination), IgG1 decreased to near zero levels whereas IgG1 + 3 remained high indicating dominant presence of IgG3 on day 600. IgG2 levels remained high, though reduced from the peak d350 levels, and with more variability within the group. On day 600, the dominant subclasses were IgG3 and IgG2 (Fig. 6a). Similar changes in the IgG subclass distribution profile were observed for the low dose group as well with each vaccination, resulting in a near zero level of IgG1 on day 600 while retaining high levels of IgG3 and IgG2 antibodies (Fig. 6b).

Antibody levels (expressed in absorbance units at 1:500 sera dilution) of different subclasses of each animal on day 600 were analyzed against corresponding TRA to assess any correlation with functional activity (Fig. 6c–e). These analyses showed strong correlations between the TRA and IgG2 (Spearman *r* = 0.903; *p* = 0.0008) (Fig. 6c) as well as TRA and IgG1 + 3 (Spearman *r* = 0.7576; *p* = 0.0149) (Fig. 6e) while a poor correlation with IgG1 (Spearman *r* = 0.333; *p* = 0.3487) (Fig. 6d). Since IgG1 on day 600 is near zero for both high-dose and low-dose groups, IgG1 + 3 values effectively correspond to IgG3. It appears that IgG3 and IgG2 are the main contributors to the long-term functional activity of Pfs230D1-EPA/ALFQ.

## Discussion

Transmission-blocking vaccines will be invaluable tools in the effort to interrupt malaria transmission. An effective TBV with activity that persists across a malaria season or more is necessary for it to be effective in the field. In this study we have explored EPA conjugates of two different TBV antigens, Pfs25 and Pfs230D1, for their ability to reduce transmission. This study carried out in Rhesus macaques over a 600-day period demonstrates that durable and effective transmission reducing activity is achievable with conjugate vaccines and potent adjuvants. Both Pfs25 and Pfs230D1 conjugates formulated with ALFQ generated strong functional antibody responses against the respective antigens, after two vaccinations. Pfs25 has been extensively studied in preclinical and clinical studies using various vaccine technology platforms whereas Pfs230 has only recently been explored as a TBV candidate.

A previous study in mice that compared various TBV antigens delivered by viral vector expression found Pfs230 and Pfs25 to have similar transmission reducing activity^31^. In a recent study Pfs25 was compared with an extended domain 1 of Pfs230 (Pfs230c1) presented on a liposomal nanoparticle platform consisting of a TLR4 agonist showed higher functional activity for Pfs25 compared to Pfs230 in mice as well as in rabbits^30^. Our earlier studies on EPA conjugates of Pfs25 and Pfs230D1 in mice, have not shown a clear distinction between these two conjugates in their functional activity^36^. However, in a recent study we observed that Alhydrogel^®^ formulated Pfs230D1-EPA conjugate had superior TRA activity in Rhesus macaque compared to Pfs25-EPA and this activity recapitulated the activity of these conjugates in humans^35^. The present study in Rhesus further validates this observation using the more potent adjuvant ALFQ and demonstrates a clear difference in functional activity between EPA conjugates of Pfs25 and Pfs230D1. The Pfs230D1 conjugate shows higher functional activity in Rhesus compared to Pfs25 conjugate, at equivalent vaccine doses.

Durable antibody response that extends across at least one season will be necessary for a TBV to be effective for implementation. For Pfs25 as well as Pfs230D1, two vaccinations were insufficient to provide durable immune responses with functional activity. Antibody levels decreased rapidly after the second vaccination at a similar rate for all the groups, accompanied with a drop in functional activity. For the Pfs25 conjugate, even at peak antibody levels on day 84 (2 weeks post vaccination), TRA was only ~80% compared to 100% for Pfs230D1 group at the same dose, clearly demonstrating superior functional activity of Pfs230D1 conjugate.

Repeat vaccinations can induce antibodies that may provide higher functional activity^46^. Since Pfs25 conjugates did not generate high functional activity after two vaccinations, they were not studied further. Groups that received Pfs230D1 were vaccinated a third time at the same dosages as previously received and induced similar peak antibody levels to the 2nd vaccination. However, 3rd vaccinations appeared to generate a more durable immune response as antibody levels decreased more slowly than was obsereved after 2nd vaccination, doubling the antibody decay half-life (17.6 days vs 36.1 days for high-dose group and 17.8 days vs 33.3 days for low-dose group). Functional activity of the immune sera also was maintained at a high level until day 600 with 4/5 from the high dose group and 3/5 from the low dose group retaining >80% TRA.

We examined whether the increase in durability of functional activity can be attributed to changes in antibody avidity. Avidity measurements carried out using sera from each animal at various time points showed an increase in avidity after each vaccination and a strong correlation with transmission reducing functional activity. Increase in avidity has been observed previously in humans upon vaccination with Outer Membrane Vesicle (OMV) vaccine against Meningococcal B and with malaria vaccine (RTS,S/AS01_E_)^46–48^. Avidity increased substantially after the 2nd vaccination and increased further on 3rd vaccination for both high and low dose groups. Avidity after 2nd vaccination remained high until the 3rd vaccination, a period during which antibody level decayed substantially. A similar phenomenon was observed with OMV vaccination and was attributed to affinity maturation occurring after the previous vaccinations^46^. A similar phenomenon might be happening here as well. Increase in serum avidity with each subsequent vaccination indicates that a larger proportion of antibody generated by each vaccination may have higher affinity and functional activity. These high affinity antibodies may contribute to the overall higher serum avidity as well as functional activity and can prolong the serum functional activity. Proportion of these antibodies can be expected to decrease over time due to the natural elimination of the circulating antibody and reach a level below that is required for measurable functional activity.

Anti-Pfs230 antibody subclass distribution showed a qualitatively different profile from what was observed for Alhydrogel^®^ formulated Pfs230D1-EPA in Rhesus macaque, where we observed an exclusively IgG1 response after 2nd and 3rd vaccinations^35^. This study showed Pfs230D1-EPA formulated with ALFQ induced all four IgG subclasses (IgG1-4) and IgM in measurable amounts after each vaccination, indicating a markedly different adjuvant effect. ALFQ contains TLR4 agonist which can induce a Th-1 skewed immune response as opposed to Th-2 response generated by Alhydrogel^®^. The dominant subclasses after the 2nd vaccination (day 84) are IgG1 and IgG3, which are both highly effective in complement activation and contribute to Pfs230 antibody mediated transmission-blocking activity. It is interesting that subsequent vaccination (day 350) alters this profile with a substantial increase in IgG2 for both high and low dose groups. At peak titers immediately after both vaccination doses, functional activity of immune sera was high presumably due to the overall high level of antibody titer. On day 600, 40 weeks after the last vaccination, when the immune response is in a steady state, we observed a mixed Th1/Th2 response where the dominant subclass are IgG3 and IgG2. At this time point we observed a strong correlation between the functional activity (% TRA) and level of IgG2 in sera, expressed in absorbance units at 1:500 dilution of sera, while also maintaining strong correlation with IgG1 + 3. Since IgG2 in Rhesus or humans is not a strong activator of complement^52^, this may indicate that antibody mediated effector functions other than complement dependent cytotoxicity also may contribute to the functional activity of Pfs230D1-EPA/ALFQ vaccine. In fact, in an earlier study of sera obtained from vaccination with Pfs230D1-EPA formulated with Alhydrogel^®^, we demonstrated that heat inactivation of complement reduced but did not completely eliminate the functional activity Rhesus and some human antisera, in spite of consisting exclusively of complement fixing IgG1 and IgG3 antibody subclasses.^35^. Additionally, a Pfs230D1 specific mAb, 4F12, showed transmission reducing activity in the presence as well as in the absence of human complement, although the activity was significantly higher in the presence of complement^28,53^. IgG2 may participate through epitope neutralization, ADCC or complement activation through multivalent binding to the high-density antigen present on the parasite surface^54,55^.

Antibody avidity as well as antibody subclass distribution may play a role in the IgG mediated functional activity after antigen exposure^51,56,57^. A recent study compared the changes in the IgG subclass levels and antibody avidity of serum antibody developed against 18 different blood stage malaria antigens in individuals during high infection period to that at low infection period^58^. This analysis has shown that IgG subclass distribution is dependent on the specific antigen but is generally dominated by cytophilic antibodies IgG1 and IgG3. Avidity of antibody increased after infection primarily due to loss of low avidity antibody from the total pool. Though the role of antibody avidity in antibody function and longevity is not fully understood, it is increasingly being recognized as an important parameter to be evaluated for understanding antibody mediated effector functions^59^. There is very limited information on the avidity or subclass distribution of Pfs230 antibodies generated due to infection or vaccination in humans or in non-human primates. This study has demonstrated that serum avidity increased with repeated vaccinations of Pfs230D1-EPA with ALFQ adjuvant. It has also demonstrated that vaccination induced a mixed Th1/Th2 immune response with high levels of IgG1 + 3 and IgG2 that also strongly correlated with transmission reducing activity. Thus the combination of high avidity and mixed Th1/Th2 antibody response may have contributed to strong transmission-blocking functional activity that lasted for at least 9 months after 3rd vaccination. It is likely that the ALFQ adjuvant played a significant role in directing this immune response. While both antibody avidity as well as the IgG subclass distribution may contribute to the durable functional activity observed, it is difficult to delineate their individual contributions from the current study.

Non-human primates, especially the Rhesus macaque, have been considered a good animal model to evaluate the safety and efficacy of human therapeutics and vaccines, due to their phylogenetic closeness to humans^60,61^. Nevertheless, the effects of vaccine-induced changes to the immunoglobulin repertoire and their functional implications are not clearly understood. Though there appears to be a nominal correspondence between IgG subclasses in human and macaque, significant differences exist in their structures as well as receptor structures^61,62^. This might impede a direct extrapolation of functional activity in nonhuman primates to that in humans. For example, IgG1 and IgG3 are most active in inducing effector functions such as ADCC and CDC in humans through their strong interaction with FcγRs and C1q, whereas IgG2 and IgG4 are less effective due to weaker binding to the receptors^63^. But in macaque, all four subclasses (IgG1-4) bind various FcγRs efficiently and induce potent ADCC and CDC^64^. Since effector functions mediated by IgG2 as well as IgG4 are species dependent, caution must be observed in extrapolating results from macaque to humans.

There have been a number of studies in Rhesus that have evaluated the IgG subclass profile induced by vaccination with protein subunit antigens in different adjuvants including ALFQ^35,46,50,65–67^. While most of these studies, either with Alum or emulsions^66^ as adjuvants, resulted in Th2 responses with IgG1 as dominant subtype, ALFQ induced a mixed Th1/Th2 response^50^. Pfs230-EPA, when formulated in Alhydrogel, yielded a Th2-like response dominated by IgG1 in both macaque and humans^35^. Whereas in ALFQ, this conjugate induced a mixed Th1/Th2 response evidenced by the IgG subclass distribution. It will be valuable to assess whether ALFQ induces a mixed Th1/Th2 response in humans and whether this has any impact on the transmission-blocking activity of this vaccine in humans.

## Methods

### Animals

Animal studies were performed according to protocols approved by the NIAID and NIH Animal Care and Use Committee. All procedures were done in accordance with the Guide for the Care and Use of Laboratory Animal Reports NIH 85-23. For vaccination studies, Rhesus macaque (*Macaca mulatta*) juvenile adults were randomized by age, sex, and weight, and were maintained in an AAALAC accredited NIAID facility. Vaccinations were performed on days 0, 70, and 322 by intramuscular injection in a volume of 0.5 mL Pfs25-EPA/ALFQ or 0.5 mL Pfs230D1-EPA/ALFQ in the leg, alternating legs for each injection.

### Antigens and conjugates

The carrier protein recombinant Exoprotein A (EPA) of *Pseudomonas aeruginosa* (molecular weight, 66,983 Da) was expressed in *E*. *coli*. Recombinant antigens Pfs25 (mol. Wt. 18,735) and Pfs230D1 (amino acids Ser^542^- Gly^736^ of domain-1 of Pfs230 with mol. wt. 21,854) were based on *P*. *falciparum* 3D7 allele sequence and were codon optimized and produced in *P*. *Pastoris*, as described previously^28^. Pfs230D1 and Pfs25 were conjugated separately to the carrier protein, EPA, by a synthetic procedure involving the following steps as previously described^20^: (a) modification of EPA with maleimide-containing linkers by treatment with *N*-(ε-maleimidocaproyloxy)succinimide (EMCS); (b) thiolation of antigen by treatment with *N*-Succinimidyl S-Acetylthioacetate (SATA) followed by deprotection of thiol by treatment with hydroxylamine; (c) conjugation between thiolated antigen and maleimide modified carrier; and (d) purification by size exclusion chromatography. Pfs230D1-EPA conjugate used in this study has an average molecular weight of 357 kDa (mol. wt. distribution: 140–820 kDa) and a molar ratio of 3.69 (antigen/carrier) This conjugate has a composition of 54.6% Pfs230D1 and 45.4% EPA. Pfs25-EPA conjugate has an average molecular weight of 373 kDa (mol. wt. distribution: 60–800 kDa), a composition of 48.7 % Pfs25 and 48.7% EPA and a molar ratio of 3.4 (antigen/carrier).

### Adjuvant formulation

An adjuvant, Army Liposome Formulation containing QS-21 (ALFQ) was prepared by Walter Reed Army Institute of Research at 2X concentration and was mixed 1:1 (v/v) with Pfs230D1-EPA conjugate or with Pfs25-EPA conjugate at room temperature and the formulation was administered to animals. Briefly, ALFQ is a liposome formulation comprising saturated phospholipids, dimyristoyl phosphatidylcholine (DMPC) and dimyristoyl phosphatidylglycerol (DMPG); cholesterol (Chol), and two adjuvants, synthetic monophosphoryl lipid A (3D-PHAD^®^) (Avanti Polar Lipids, Alabaster, AL) and QS-21 (Desert King, San Diego, CA)^68^. QS-21 is a triterpenoid glycoside saponin derived from the bark of the *Quillaja saponaria* (soap bark) tree, found in Chile. In all, 1 mL of diluted ALFQ used in the study consisted of 6986.27 µg (10.305 mM) DMPC; 788.73 µg (1.145 mM) DMPG; 5413 µg (14.0 mM) Cholesterol; and 200 µg 3D PHAD^®^ and 100 µg QS-21. Final delivery dose of 0.5 mL contained 100 ug 3D-PHAD^®^ and 50 ug QS-21 for each animal. The composition and dose of this adjuvant is same as that is currently used in clinical trials.

### Standard membrane feeding assay

Functional activity of immune sera were assayed by an ex vivo standard membrane feeding assay (SMFA) in terms of their transmission-blocking activity (TBA, reduction in mosquito infection prevalence) and transmission reducing activity (TRA, reduction in mosquito infection intensity) as described previously^35^. In vitro 14–16-day-old gametocyte culture of *P. falciparum* (NF54 line) was diluted with washed O + red blood cells (RBCs) from a malaria naïve donor (Interstate Blood Bank, Memphis, Tennessee) to achieve 0.12 ± 0.05% concentration of Stage V gametocytes. For each sample, 100 µL of the diluted culture was mixed with 160 µL of test serum (60 μL of test sera mixed with 100 μL of a pool of naïve human AB^+^ sera). All samples were immediately fed to pre-starved (~24 h) 3–8-day-old *Anopheles stephensi* (Nijmegen strain) mosquitoes through a membrane feeding device maintained at 40 °C. Test sera were not heat-inactivated. Mosquitoes, after feeding, were maintained for 8 days at 27 °C and 80% humidity conditions to allow for the development of parasites. Mosquitos were then dissected, midguts were stained with 0.05% mercurochrome and the number of oocysts on each midgut were counted. The TBA and TRA were calculated by the following formulas:

1 $${{{\mathrm{TRA}}}} = 100\;{{{{{\mathrm{x}}}}}}\left( {\frac{{{{{\mathrm{Mean}}}}\;{{{\mathrm{Oocyst}}}}\;{{{\mathrm{Number}}}}_{{{{\mathrm{neg}}}}\;{{{\mathrm{ctrl}}}}} - {{{\mathrm{Mean}}}}\;{{{\mathrm{Oocyst}}}}\;{{{\mathrm{Number}}}}_{{{{\mathrm{test}}}}}}}{{{{{\mathrm{Mean}}}}\;{{{\mathrm{Oocyst}}}}\;{{{\mathrm{Number}}}}_{{{{\mathrm{neg}}}}\;{{{\mathrm{ctrl}}}}}}}} \right)$$

and

2 $${{{\mathrm{TBA}}}} = 100\;{{{{{\mathrm{x}}}}}}\left( {\frac{{{{{\mathrm{Mean}}}}\;{{{\mathrm{No.Inf.Mosquito}}}}_{{{{\mathrm{neg}}}}\;{{{\mathrm{ctrl}}}}} - {{{\mathrm{Mean}}}}\;{{{\mathrm{No.Inf.Mosquito}}}}_{{{{\mathrm{test}}}}}}}{{{{{\mathrm{Mean}}}}\;{{{\mathrm{No.Inf.Mosquito}}}}_{{{{\mathrm{neg}}}}\;{{{\mathrm{ctrl}}}}}}}} \right)$$

where the negative control (neg ctrl) feed used pre-vaccination sera pool.

### ELISA

ELISA was done as described previously^35^. Plates (Immulon 4 HBX flat bottom microtiter plates (Dynex Technologies) were coated with 1 μg/ml of Pfs25 or Pfs230D1 in a volume of 100 μL per well in carbonate coating buffer (pH 9.6) overnight at 4 °C. Wells were blocked with 5% skim milk in TBS blocking buffer in a volume of 320 μL per well for 2 h. Samples were serially diluted in TBS/5% milk and added to wells in triplicate in a volume of 100 μL per well followed by incubation at room temperature for 2 hours. After washing the plates four times, alkaline phosphatase labeled goat anti-monkey secondary antibody (Seracare Life Sciences) was added in a volume of 100 μL per well and incubated at room temperature for 2 h. After 4 washes, 100 μL each of phosphatase substrate (dissolved tablets, Sigma) were added to each well. Plates were incubated for 20 min before optical densities were measured with a Spectramax 340PC (Molecular Devices). Each ELISA plate contained an internal serum standard from which a four-parameter curve was calculated with Softmax software. ELISA Units were assigned to test samples based on the sera dilution that gave an OD of 1.0, adjusted to the internal standard.

To determine Pfs230 specific IgG subclasses (on days 0, 84, 350, and 600), detecting antibodies specific for Rhesus IgG1, IgG1 + IgG3, IgG2, and IgG4 were obtained from the NHP Resource Reagent, US (Supplementary Table 5).

### Antibody avidity measurements

To deactivate the complements and lipid contents, sera were heated at 56 °C for 45 min followed by centrifugation at 16,000 × *g* at 4 °C for 20 min and the supernatants were collected and used in the Biacore avidity assay. The subsequent procedure was conducted with a Biacore 4000 system as previously described^50,69^. The immobilizations were completed in 10 mM Hepes and 150 mM NaCl pH 7.4 using a standard amine coupling kit. The CM5-S series chip surface was activated with a 1:1 mixture of 0.4 M 1-ethyl-3-(3-dimethylaminopropyl) carbodiimide hydrochloride (EDC) and 0.1 M *N*-hydroxysuccinimide (NHS) for 600 s. Then 5 μg/mL or 20 μg/mL Pfs230D1 protein in 10 mM sodium acetate pH 4.0 were immobilized to spots 1, 2, 4, and 5 to each flow cell of the CM5 sensor chip. The density was immobilized in the range of 400–600 RU (low density) and 600–850 RU (high density). Spot 3 in each flow cell was left unmodified to serve as a reference. The immobilized surface was then deactivated by 1.0 M ethanolamine-HCl pH 8.5 for 600 s. Following the surface preparation, the heat-inactivated sera samples were diluted 1:50 in 10 mM Hepes, 300 mM NaCl, 0.005% Tween-20, pH7.4 running buffer and injected onto the antigen-immobilized surface for 250 s followed by dissociation for 2000 s. The bound surface was then enhanced with a 250-s injection of 30 µg/mL of the secondary antibody goat anti-monkey IgG. To regenerate the bound surface, 175 mM HCl was injected twice for 60 s. Four replicates for each sample were collected at rate of 10 Hz, with an analysis temperature of 25 °C. All sample injections were conducted at a flow rate of 10 μL/min. Data analysis was performed using Biacore 4000 Evaluation software 4.1 with double subtractions for an unmodified surface and buffer for the blank. Fitting was conducted using the dissociation mode integrated with Evaluation software 4.1.

### Statistical analysis

Statistical analyses were carried out using Prism v7.0 by GraphPad Software, Inc. IC50 values were estimated using a 4-parameter curve fit of the log(EU) vs %TRA for each monkey for the d84 and d168 SMFA data combined for Fig. 2b and d434 and d600 data combined for Fig. 4b. Statistical significance of the difference in avidity between d84 and d350 groups in Supplementary Fig. 4a, b were done by Wilcoxon matched-pairs signed rank analysis and Mann–Whitney test. Antibody decay half-life after second and third vaccinations respectively were determined by analyzing the data between d84 and d280 and between d350 and d600, using Prism v7.0 with an exponential decay model. General estimating equations were used to model differences in ELISA units in animals that received the high dose or low dose Pfs230-EPA ALFQ vaccine (Figs. 1b and 3). Pfs230 ELISA titers were log-transformed and used as the outcome variable, while group (low dose or high dose) and study day were used as the predictor variables.

### Reporting summary

Further information on research design is available in the Nature Research Reporting Summary linked to this article.

## Supplementary information

Supplementary Information

Reporting Summary

## Data Availability

The datasets generated during and/or analyzed during the current study are available from the corresponding author on reasonable request.
