# Supplementary material for: Malaria transmission-blocking conjugate vaccine in ALFQ adjuvant induces durable functional immune responses in rhesus macaques
Source: NPJ Vaccines. 2021 Dec 9;6:148. doi: 10.1038/s41541-021-00407-3 (PMC8660773; doi:10.1038/s41541-021-00407-3)
Supplement: Supplementary file 1 — Supplementary Information [file 41541_2021_407_MOESM1_ESM.pdf]

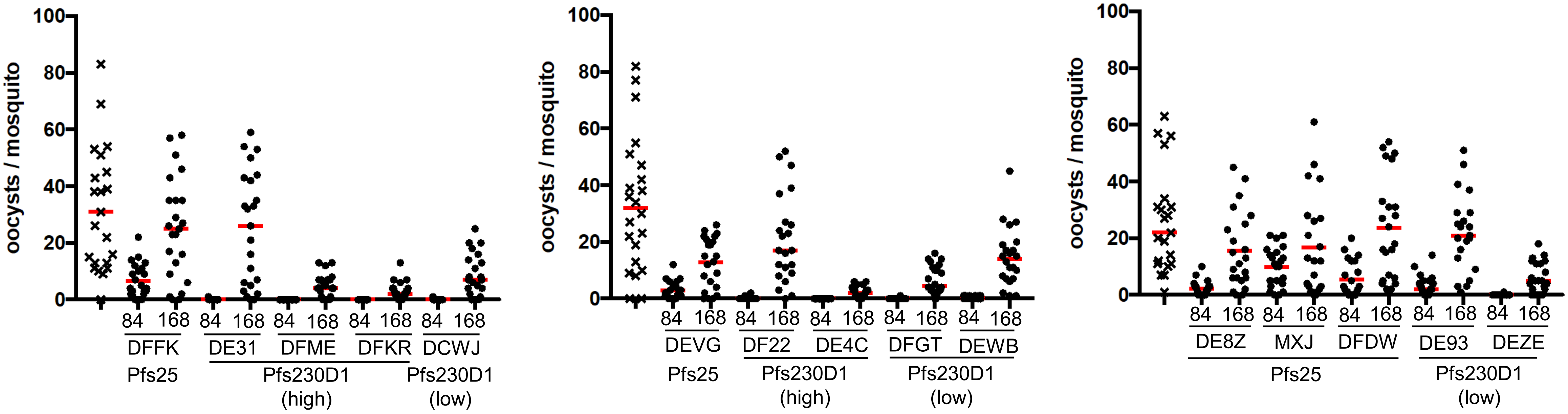

Supplementary Figure 1: SMFA of sera from vaccinated monkeys. Oocyst burden in mosquitos fed on sera drawn on days 84 and 168 from individual monkeys vaccinated with Pfs25-EPA or Pfs230D1-EPA, with the animal IDs shown on the x-axis. Each graph is the result of a single SMFA. Each data point represents the number of oocysts in one mosquito; red lines denote arithmetic mean; x symbols are a pool of sera from all monkeys before the first vaccination.

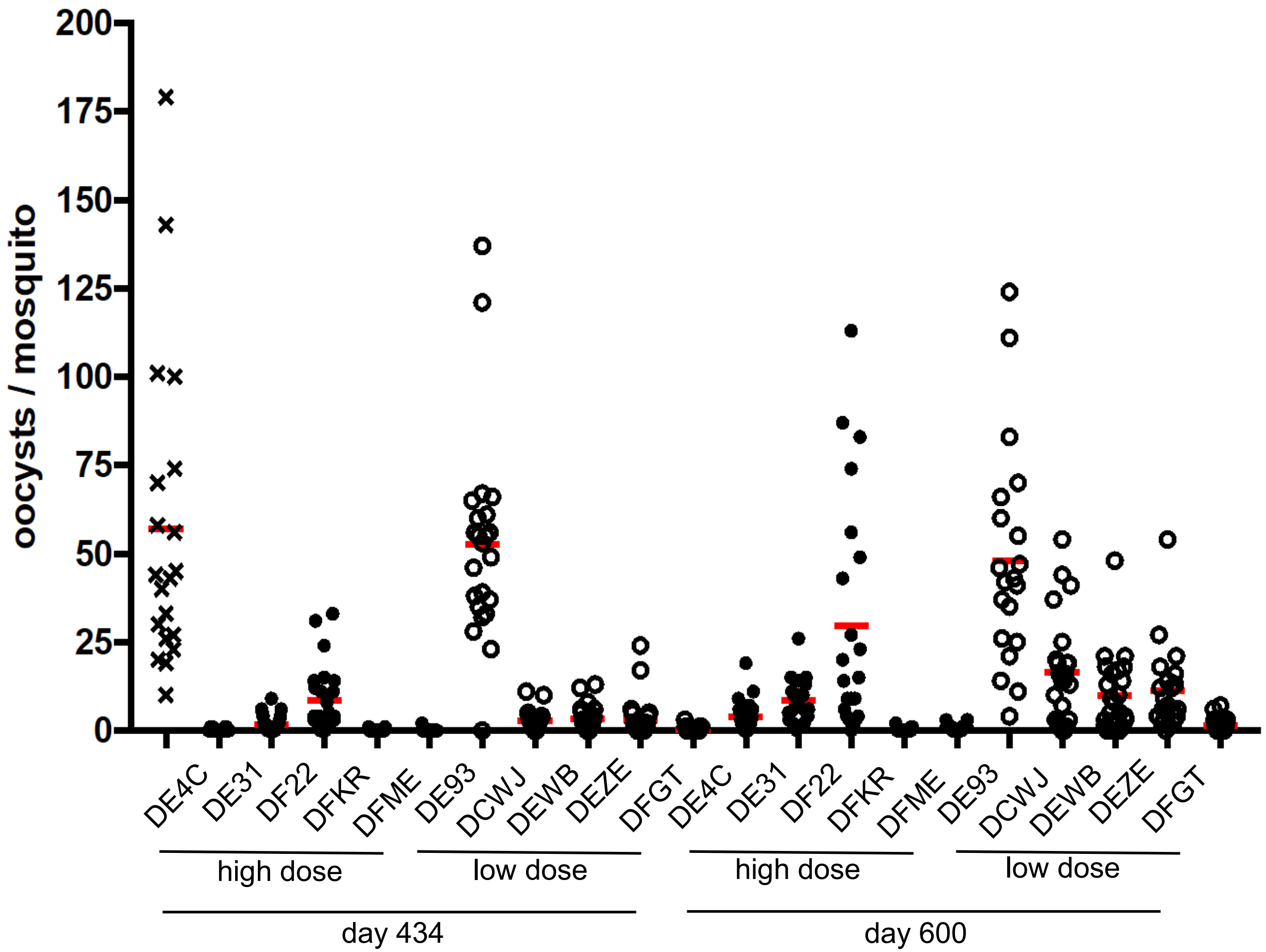

Supplementary Figure 2: SMFA of sera from vaccinated monkeys. Oocyst burden in mosquitos fed on sera drawn on days 434 and 600 from individual monkeys vaccinated with Pfs230D1-EPA conjugate at high and low doses, with the animal IDs shown on the x-axis. Each graph is the result of a single SMFA. Each data point represents the number of oocysts in one mosquito; red lines denote arithmetic mean; x symbols are a pool of sera from all monkeys before the first vaccination.

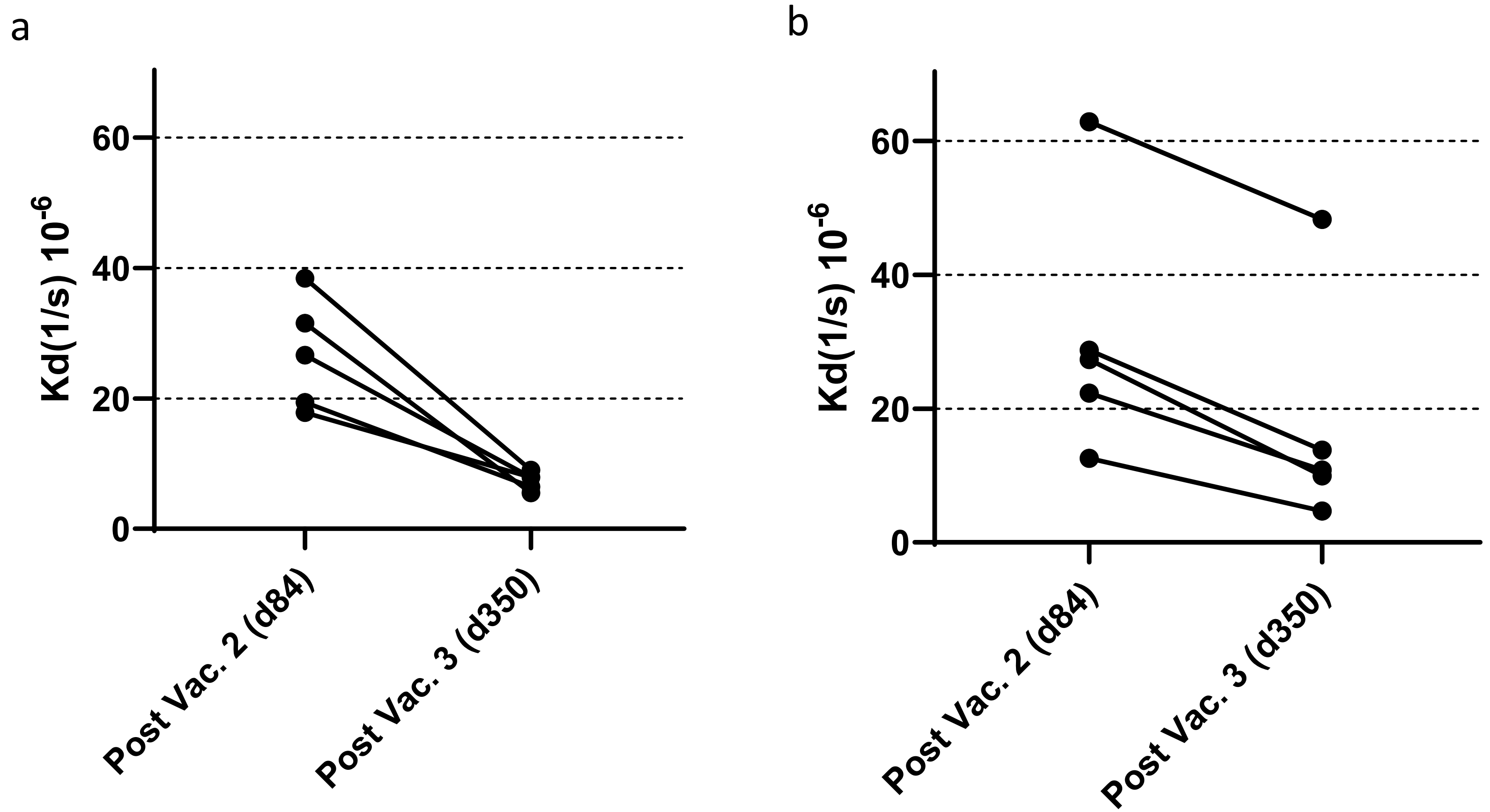

Supplementary Figure 3: Antibody avidity, presented as dissociation constant Kd, of sera from animals immunized with Pfs230D1-EPA conjugate. a) Change in avidity of standard dose group from post vaccination 2 (d84) to post vaccination 3 (d350). b) Change in avidity of low dose group from post vaccination 2 (d84) to post vaccination 3 (d350)

Supplementary Table 1: SMFA after 2 vaccinations

		Day 84				Day 168			
	Rhesus identity	mean oocysts	%TRA	infected/ dissected	%TBA	mean oocysts	%TRA	infected/ dissected	%TBA
**Assay #1**									
**Pre-vaccination sera pool**		36.0	--	22/23	--	36.0	--	22/23	--
**Pfs25-EPA**	DFFK	6.5	82.0	19/23	13.6	25.1	30.2	21/23	4.6
**Pfs230D1-EPA (high)**	DE31	0.04	99.9	1/23	95.5	25.0	30.4	21/23	4.6
	DFME	0	100	0/22	100	4.6	87.2	17/23	22.7
	DFKR	0	100	0/23	100	2.9	91.9	18/25	18.2
**Pfs230D1-EPA (low)**	DCWJ	0.04	99.9	1/23	95.5	8.2	77.5	18/25	18.2
**Assay #2**									
**Pre-vaccination sera pool**		33.3	--	20/22	--	33.3	--	20/22	--
**Pfs25-EPA**	DEVG	2.9	91.4	17/22	15.0	12.8	61.7	19/22	5.0
**Pfs230D1-EPA (high)**	DF22	0.1	99.6	2/22	90.0	20.9	37.4	21/22	0
	DE4C	0	100	0/22	100	2.3	93.2	18/23	13.9
**Pfs230D1-EPA (low)**	DFGT	0.1	99.9	1/22	95.0	6.8	79.7	20/22	0
	DEWB	0.3	99.1	7/22	65.0	14.6	56.1	22/22	0
**Assay #3**									
**Pre-vaccination sera pool**		25.9	--	21/21	--	25.9	--	21/21	--
**Pfs25-EPA**	DE8Z	2.1	91.9	13/22	40.9	15.7	38.5	20/22	9.1
	MXJ	9.9	61.8	20/22	9.1	16.7	35.4	20/22	9.1
	DFDW	5.5	78.9	16/22	27.3	23.7	8.4	22/22	0
**Pfs230D1-EPA (low)**	DE93	3.3	87.4	17/23	26.1	21.9	15.4	21/21	0
	DEZE	0.1	99.8	1/22	95.5	6.1	76.3	17/22	22.7

Supplementary Table 2: Anti-Pfs230D1 IgG titers in rhesus

	Rhesus – identity code	Day 84	Day 168	Day 322	Ratio of 84/322	Day 350	Day 434	Day 600	Ratio of 350/600
**Pfs230D1-EPA/ALFQ (high dose)**	DE31	20,517	3,611	2,191	9.4	32,965	11,452	7,285	4.5
	DFME	59,569	5,287	2,616	22.8	68,089	22,628	10,549	6.5
	DFKR	38,463	5,551	3,741	10.3	51,838	23,096	11,951	4.3
	DF22	19,177	2,527	919	20.9	13,184	3,796	2,848	4.6
	DE4C	47,825	5,131	1,287	37.2	43,038	8,506	3,528	12.2
	Geometric mean	33,645	4,243	1,909	17.6	36,638	11,408	6,209	5.9
**Pfs230D1-EPA/ALFQ (low dose)**	DCWJ	20,323	1,831	437	46.5	8,861	2,978	1,156	7.7
	DFGT	27,769	2,669	1,176	23.6	55,286	16,326	7,469	7.4
	DEWB	21,911	2,952	1,210	18.1	22,215	6,027	3,656	6.1
	DE93	4,473	781	352	12.7	3,342	1,384	592	5.6
	DEZE	41,425	3,805	1,079	38.4	14,678	3,438	2,100	7.0
	Geometric mean	18,708	2,120	750	24.9	13,980	4,255	2,083	6.7

Supplementary Table 3: SMFA after 3 vaccinations

		Day 434				Day 600			
	Rhesus identity	mean oocyst count	%TRA	infected/ dissected	%TBA	mean oocyst count	%TRA	Infected/ dissected	%TBA
**Pre-vaccination sera pool**		57.1	--	20/20	--	57.1	--	20/20	--
**Pfs230D1-EPA/ALFQ (high)**	DE31	1.9	96.7	17/28	39.3	8.3	85.4	20/21	4.8
	DFME	0.1	99.8	1/21	95.2	0.8	98.6	16/29	44.8
	DFKR	0.1	99.8	2/23	91.3	0.2	99.6	4/23	82.6
	DF22	8.6	85.0	21/24	12.5	29.6	48.1	21/22	4.5
	DE4C	0.2	99.7	4/22	81.8	4.0	93.1	20/25	20.0
**Pfs230D1-EPA/ALFQ (low)**	DCWJ	2.9	95.0	17/21	19.0	16.4	71.2	22/24	8.3
	DFGT	0.3	99.4	6/23	73.9	1.4	97.5	10/20	50.0
	DEWB	3.4	94.1	18/22	18.2	9.8	82.8	19/24	20.8
	DE93	52.7	7.6	22/23	4.3	48.1	15.8	20/20	0
	DEZE	3.1	94.5	17/27	37.0	11.3	80.1	20/21	4.8

Supplementary Table 4: Avidity of immune sera

		Kd(1/s) e-05		
	Rhesus identity	Day 29	Day 84	Day 350
**Pfs230D1-EPA/ALFQ (high dose)**	DE31	53.1	3.84	0.9
	DFME	n.d.	3.15	0.54
	DFKR	15.9	1.78	0.79
	DF22	15.0	1.94	0.643
	DE4C	95.1	2.66	0.79
**Group mean**		34.5	2.67	0.73
**Pfs230D1-EPA/ALFQ (low dose)**	DCWJ	n..d	2.73	0.997
	DFGT	42.9	1.25	0.472
	DEWB	28.9	2.87	1.38
	DE93	n.d.	6.28	4.83
	DEZE	35.6	2.23	1.08
**Group mean**		35.8	2.73	1.75

Supplementary Table 5: Reagents used for IgG subclass ELISA

Reagent	clone	Source
anti-IgG1	7H11	NHP Reagent Resource, US
anti-IgG1+IgG3	1B3	NHP Reagent Resource,US
anti- IgG2	Dio	NHP Reagent Resource,US
anti-IgG4	Tessera	NHP Reagent Resource, US
IgM	polyclonal	Jackson Immunoresearch, US
